# Supplementary material for: The ATM and ATR kinases regulate centrosome clustering and tumor recurrence by targeting KIFC1 phosphorylation
Source: Nat Commun. 2021 Jan 4;12:20. doi: 10.1038/s41467-020-20208-x (PMC7782532; doi:10.1038/s41467-020-20208-x)
Supplement: Supplementary file 1 — Supplementary Information [file 41467_2020_20208_MOESM1_ESM.pdf]

Supplementary data Figure 1

a

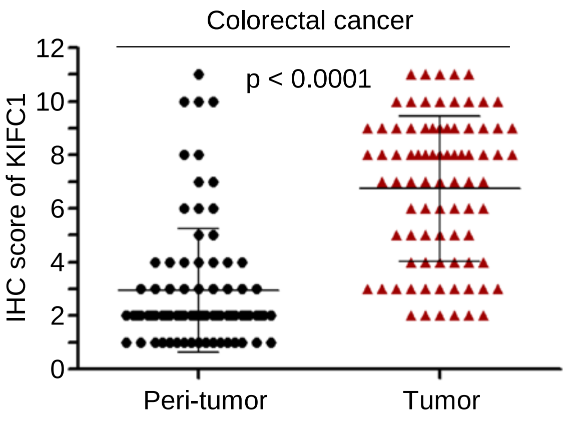

b

Colorectal cancer

IHC staining reaction (n = 83)				*P* value
	tumor			
peri-tumor	Low	Medium	High	
Low	19	32	19	< 0.05
Medium	2	2	5	
High	1	1	2	

Figure S1, Related to Figure 1. **KIFC1 is upregulated in human colorectal cancer.** (a) Scatter plot shows quantitative analysis of peri-tumor and tumor in colorectal cancer clinical samples. Data represent the mean ± SD. Two-tailed t test *p* values: p = <0.00001. (b) The level of KIFC1 expression was classified as high, medium, or low according to the staining signals in each group. The table shows the percentages of tissues with different levels of staining of KIFC1 in peri-tumor and tumor tissues. Two-tailed student t test *p* values: $p = 0.0282$. Source data are provided as a Source Data file.

Supplementary data Figure 2

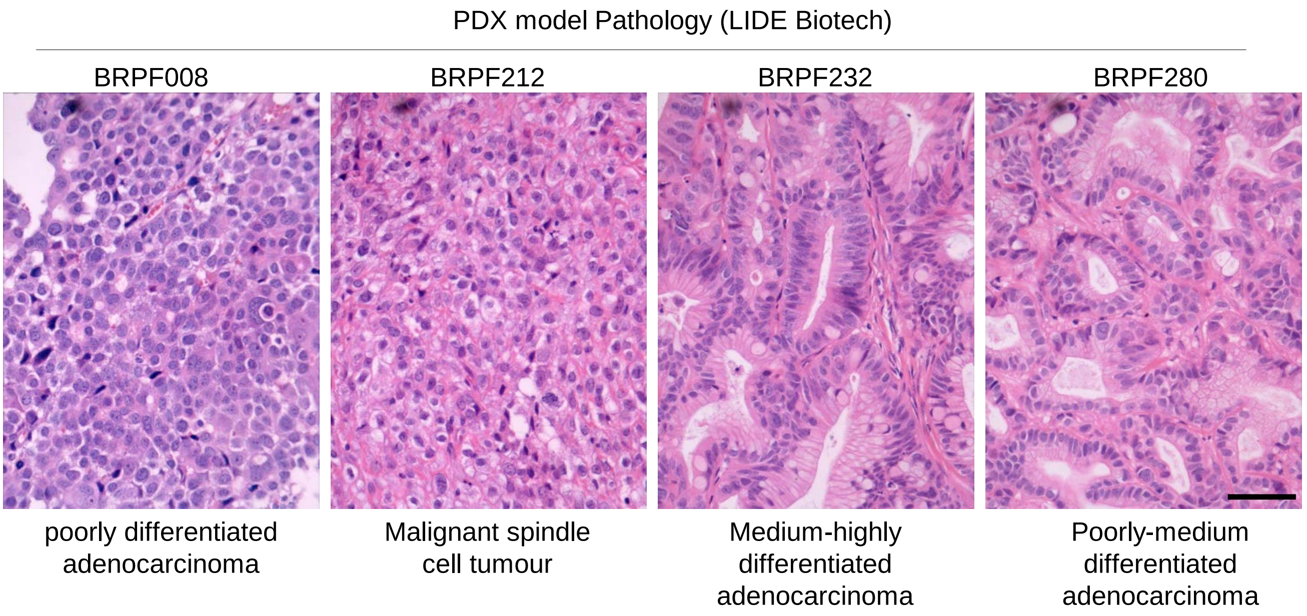

Figure S2, Related to Figure 2. The characteristic of tumor tissues from four breast cancer patient- derived xenograft models. Data are representative of stained tumor tissues (scale bar, 50 μm). Hematoxylin and eosin staining of tumor tissue sections. The data is provided by LIDE Biotech (Shanghai, China).

Supplementary data Figure 3

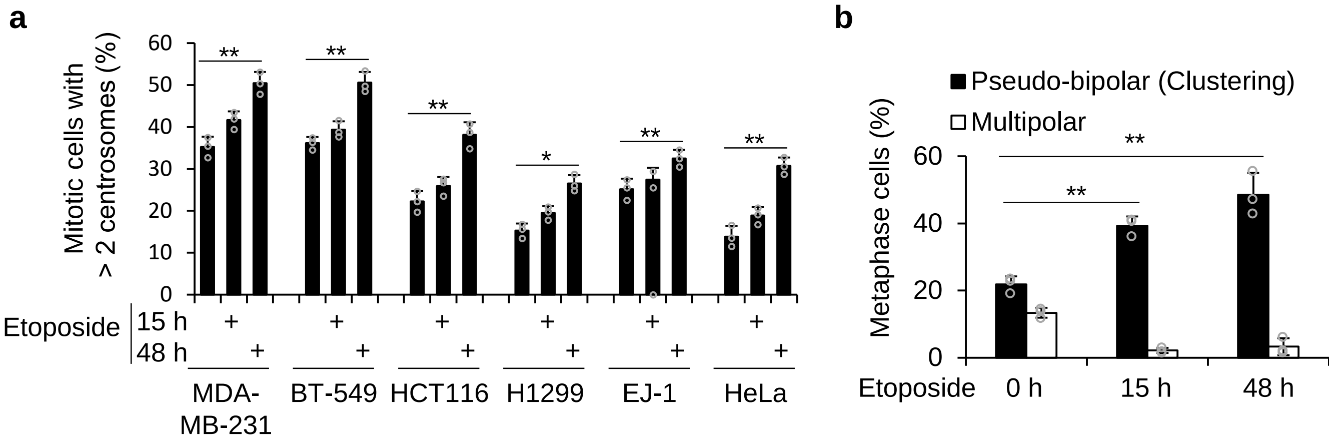

Figure S3, Related to Figure 3. **DNA-damaging treatments induce centrosome amplification and clustering.** (a) The percentage of mitotic cells that show more than two centrosomes per cell in the cell lines as indicated in response to etoposide (5 μM) for 15 h or 48 h. For each experimental condition, 100-140 cells were counted, and three independent experiments were performed. Two-tailed t test *p* values (from left to right): $p = 0.0083$, 0.0093, 0.0007, 0.0342, 0.0016, and 0.0082. *$p < 0.05$; **$p < 0.01$. (b) The percentage of pseudo-bipolar mitosis (centrosome clustering) and multipolar mitosis (non-efficient centrosome clustering) in MDA-MB-231 cells in response to etoposide (5 μM) for 15 h or 48 h. For each experimental condition, 100-167 cells were counted, and three independent experiments were performed. Two-tailed test *p* values (from left to right): $p =$ 0.0097 and 0.0098. Data show mean values ± SD of three times of independent experiments. Source data are provided as a Source Data file.

Supplementary data Figure 4

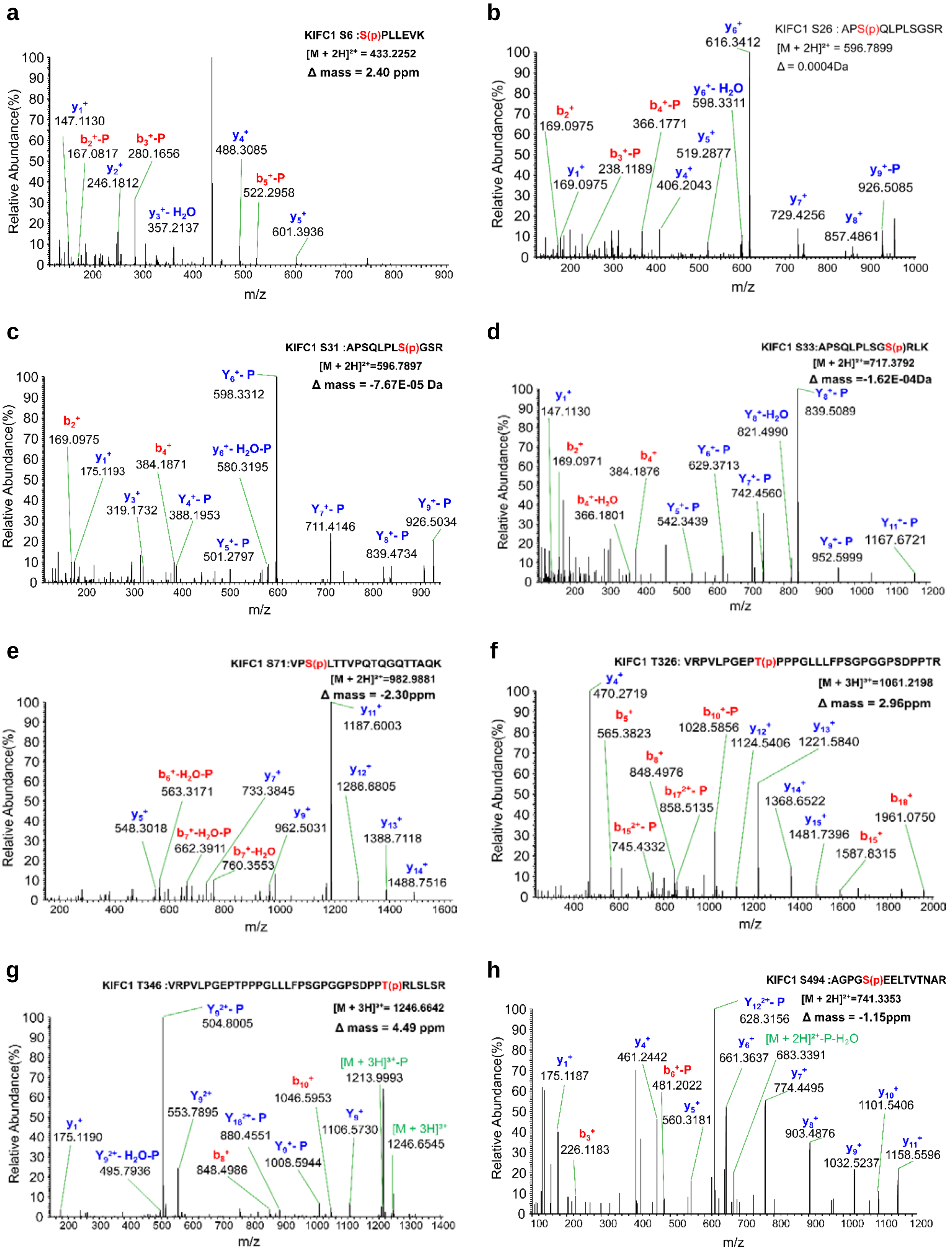

Figure S4, Related to Figure 4. Representative MS/MS spectra showing phosphorylation of KIFC1 at S6 (a), S26 (b), S31 (c), S33 (d), S71 (e), T326(f), T346(g), S494(h)

Supplementary data Figure 5

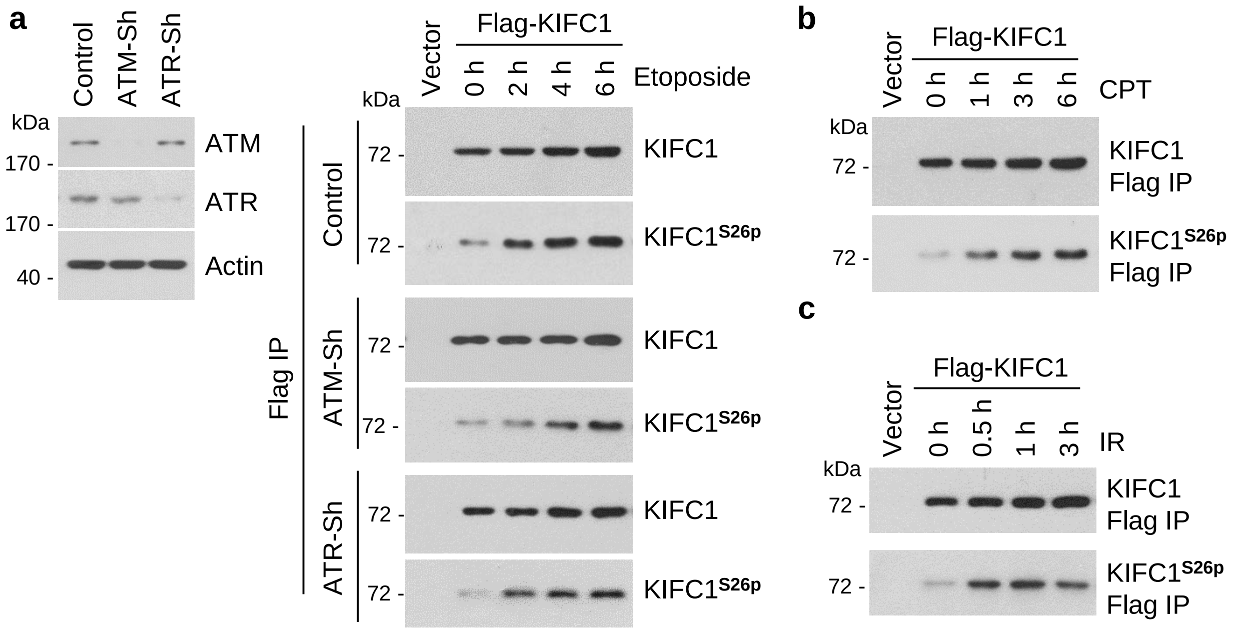

Figure S5, Related to Figure 4. **KIFC1-S26 is phosphorylated by DNA-damaging therapies.** (a-c) 293T cells were transfected with Flag-KIFC1 plasmid and treated with etoposide (20 µM) (a), camptothecin (CPT, 500 nM) (b), or irradiation (IR, 10 Gy) (c) for the indicated times, followed by immunoprecipitation with FLAG-M2 beads. The precipitated proteins were analyzed by western blotting using antibody against KIFC1 or KIFC1^{S26p}. (a) Cells were infected with ShN (Control), ATM-ShR, or ATR-ShR lentivirus for 72 h and then transfected with Flag-KIFC1 plasmid. Cell lysates were immunoblotted with antibodies against ATM, ATR, or β-actin (as the internal standard). The images are representative of 2 independent experiments. Source data are provided as a Source Data file.

Supplementary data Figure 6

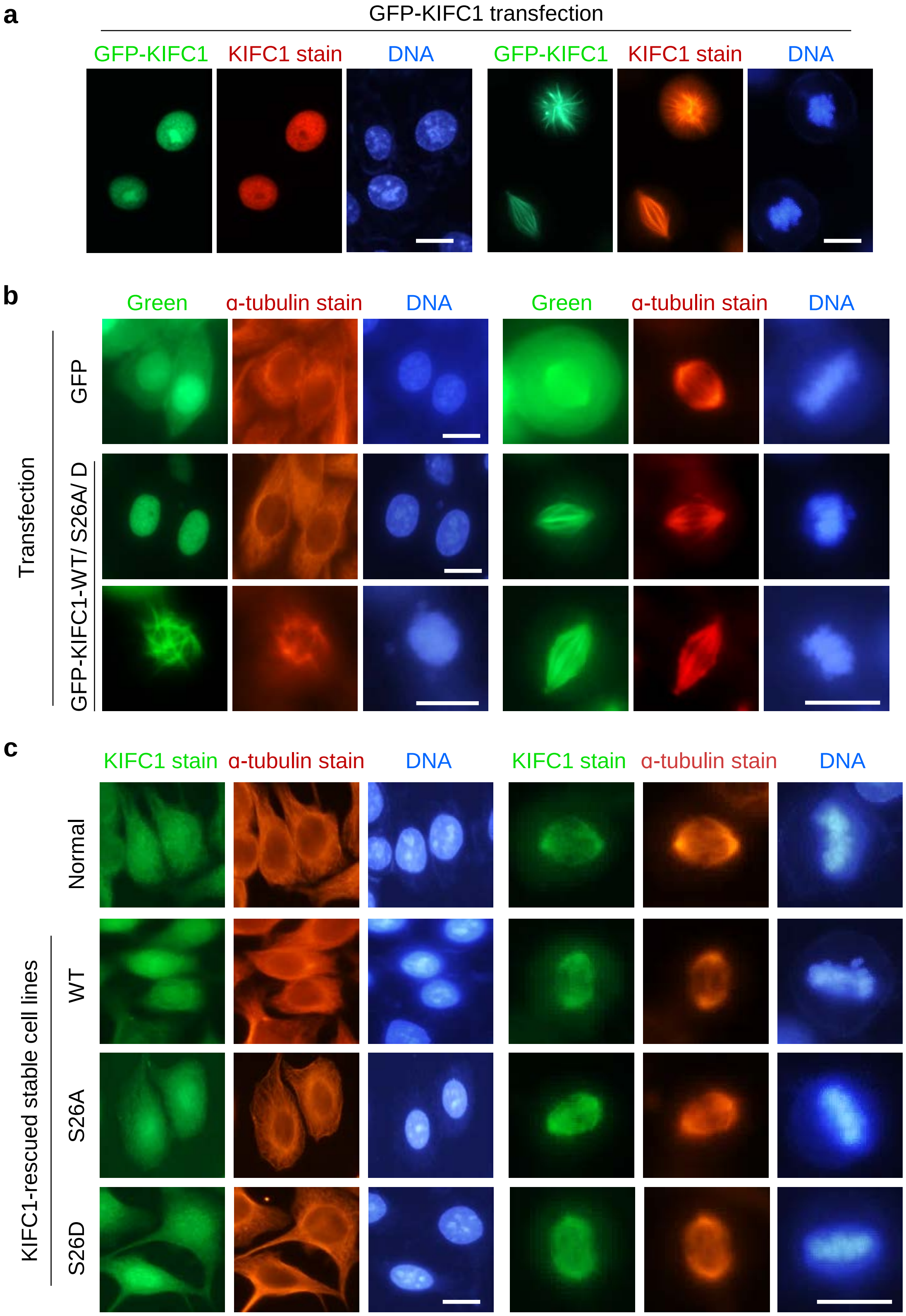

Figure S6, Related to Figure 5. The cell characterization of KIFC1-WT and mutant stable cell lines, and cells transfected with KIFC1 or mutant plasmids. (a) The specificity of the KIFC1 antibody stain (ab172620, Abcam) in MDA-MB-231 cells, which were transfected with the GFP-KIFC1 plasmid for 24 h. (b) GFP-KIFC1-WT, S26A, or S26D plasmids, which were transiently transfected into MDA-MB-231 cells respectively, led to microtubule bundles and longer spindles. (c) KIFC1-WT, S26A, or S26D stable cell lines did not show microtubule bundles and longer spindles. KIFC1 was localized in the centrosome, nucleus, and cytoplasm. Representative images showing KIFC1, α-tubulin, or DNA in the indicated cell lines (scale bar, 10 μm). Spindle poles, KIFC1, and DNA were co-stained with α-tubulin, KIFC1, and DAPI, respectively. The panels show representative images of three independent experiments.

Supplementary data Figure 7

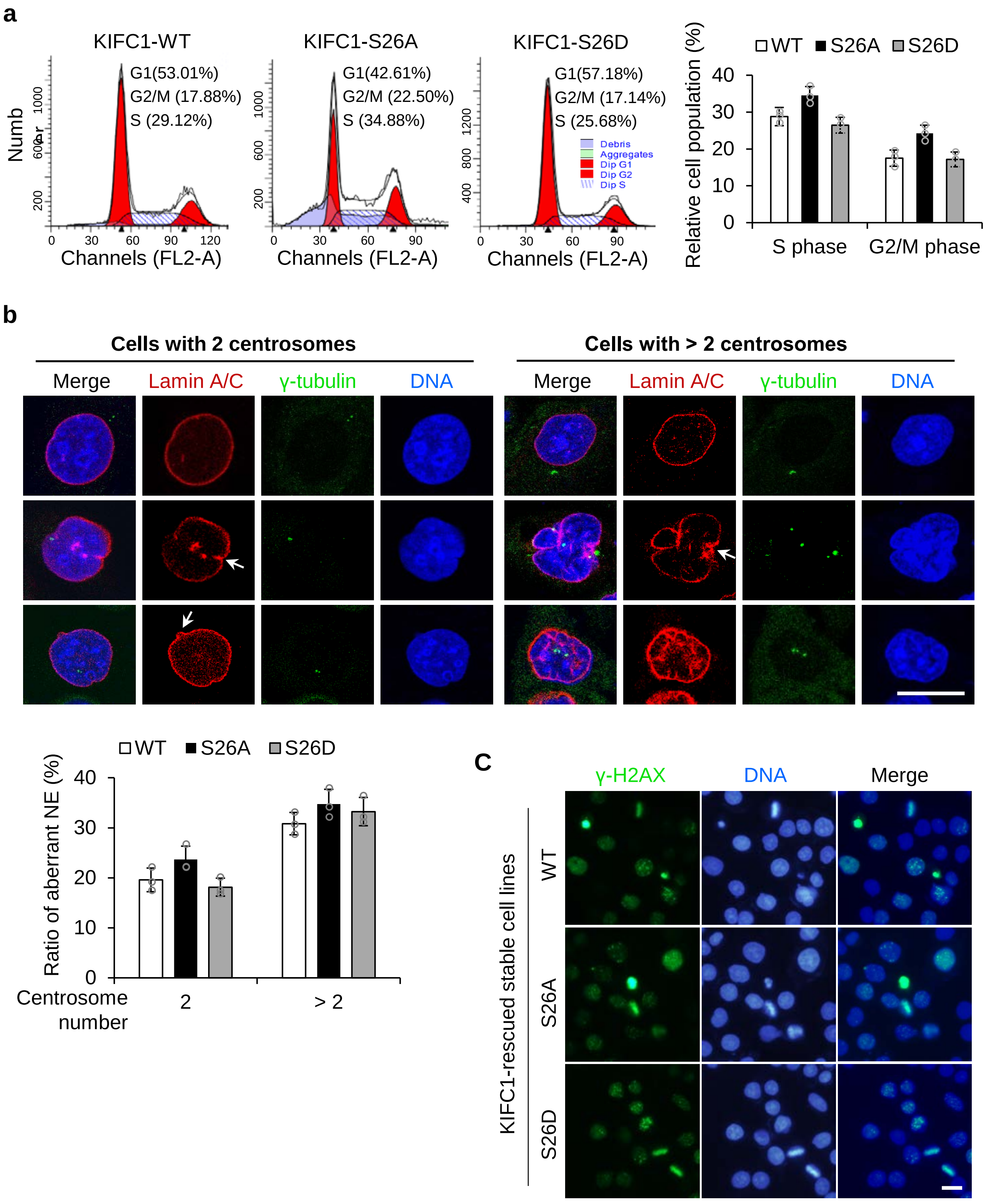

Supplementary data Figure 7, cont'd

Figure S7, Related to Figure 6. **The cell characterization of KIFC1-WT and mutant stable cell lines in cell cycle progression and aberrant nuclear membrane.** (a) KIFC1-S26A cells show slightly prolonged S and G2/M phases compared with KIFC1-WT and -S26D cells. Indicated cell lines were stained with propidium iodide (PI) and analyzed by FACS. Percentage of cells in S or G2/M phase are shown. (b) KIFC1 knockdown MDA-MB-231cell lines were rescued with KIFC1-WT, -S26A, or -S26D. The percentage of cells with aberrant nuclear envelope (NE) was recorded. There were no significant difference of aberrant NE ration between different cell lines either with 2 centrosomes or with more than 2 centrosomes. Representative images showing Lamin A/C, γ-tubulin, or DNA in the indicated cell lines (scale bar, 10 μm). White arrows indicate the invaginated, blebbed, multi-nucleated, diffused, or concaved nuclear lamina. For each experimental condition, 100-114 cells were counted, and three independent experiments were performed. (c) KIFC1-WT, S26A, and S26D mutant cells did not show serious DNA damage except some dying cells under normal culture conditions. Representative images show γ-H2AX or DNA in the indicated cell lines (scale bar, 10 μm). Statistical data presented in this figure show mean values ± SD of three times of independent experiments. The images are representative of three independent experiments. Source data are provided as a Source Data file.

Supplementary data Figure 8

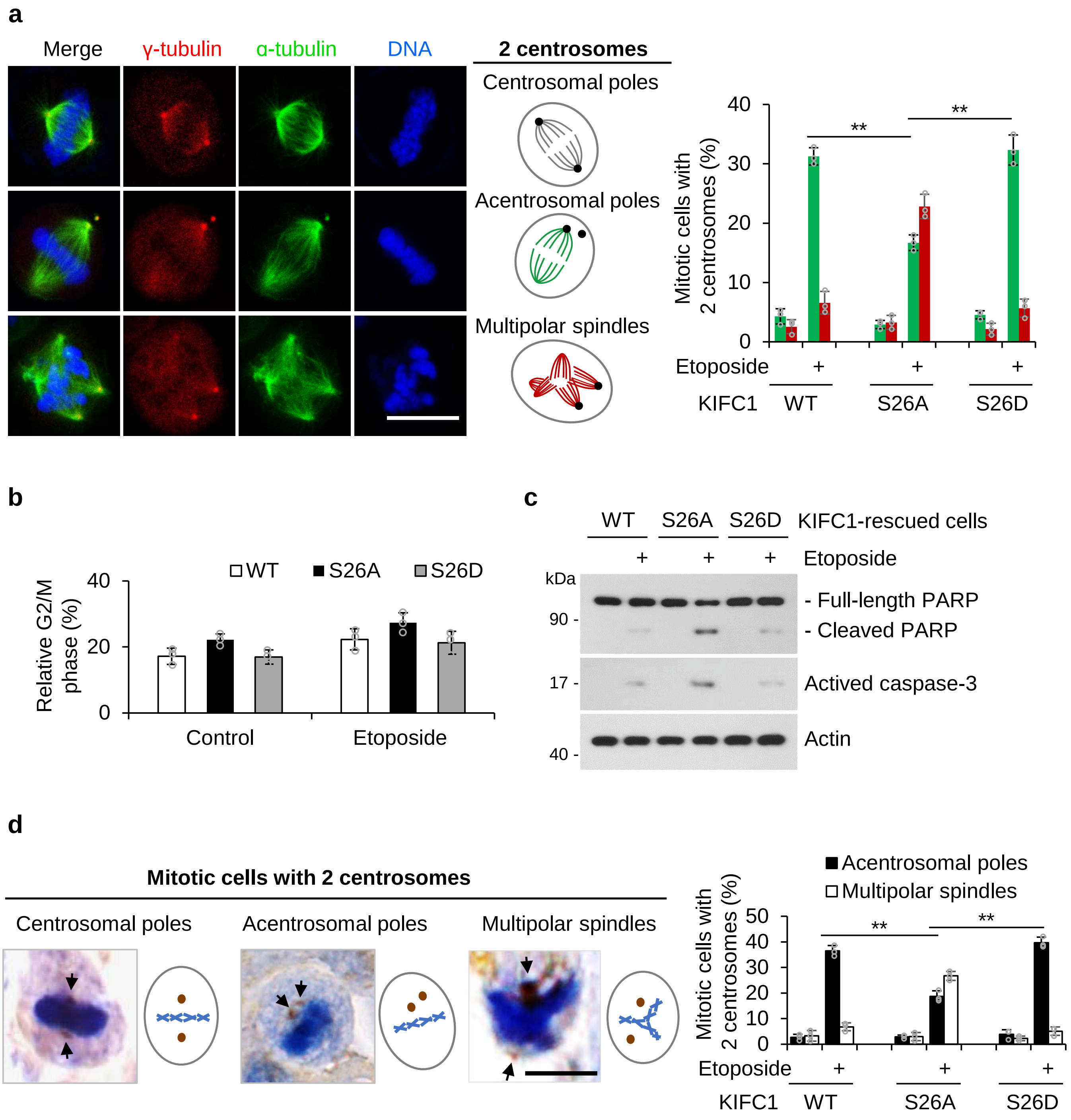

Supplementary data Figure 8, cont'd

Figure S8, Related to Figure 6. DNA damage correlates with acentrosomal spindle organization and KIFC1-S26 phosphorylation-dependence in cells with two centrosomes. (a) Representative images showing centrosomal poles, acentrosomal poles, and multipolar, fragmented spindles in KIFC1-WT and mutant stable cell lines with two centrosomes (scale bar, 10 μm). Three different patterns of spindle morphology were defined as illustrated on the right. (1) Bipolar spindles with centrosomal poles (gray spindle and bars, black centrosomes), (2) bipolar spindles with acentrosomal pole(s) and free centrosome (green spindle and bars), and (3) multipolar, fragmented spindles with centrosomal and acentrosomal pole(s) (red spindle and bars). Spindle poles, centrosomes, and DNA were co-stained with α-tubulin, γ-tubulin, and DAPI. Quantitative analyses of spindle morphologies in cells with 2 centrosomes after etoposide treatment (2 μM) for 48 h. For each experimental condition, 100-121 cells were counted, and three independent experiments were performed. Two-tailed t test *p* values (from left to right): $p = 0.0097$ and 0.0021. (b) Indicated stable cell lines were treated with etoposide for 48 h, and then stained with propidium iodide (PI) and analyzed by FACS. Percentage of cells in G2/M phase are shown. (c) Indicated stable cell lines were treated with etoposide for 48 h and then analyzed for activated caspase-3 and Poly (ADP-ribose) polymerase cleavage by western blotting. The image is representative of 2 independent experiments. (d) Representative immunohistochemical images show γ-tubulin staining with quantitative analyses of acentrosomal poles and multipolar, fragmented spindles (with 2 centrosomes) in the tumor sections of xenograft tumor samples after treatment with etoposide treatment for 4 weeks. Arrows point to the centrosomes. DNA was stained with hematoxylin. Two-tailed student t test *p* values: $p = 0.0091$ and 0.0099. Statistical data presented in this figure show mean values ± SD of three times of independent experiments. Source data are provided as a Source Data file.

Supplementary data Figure 9

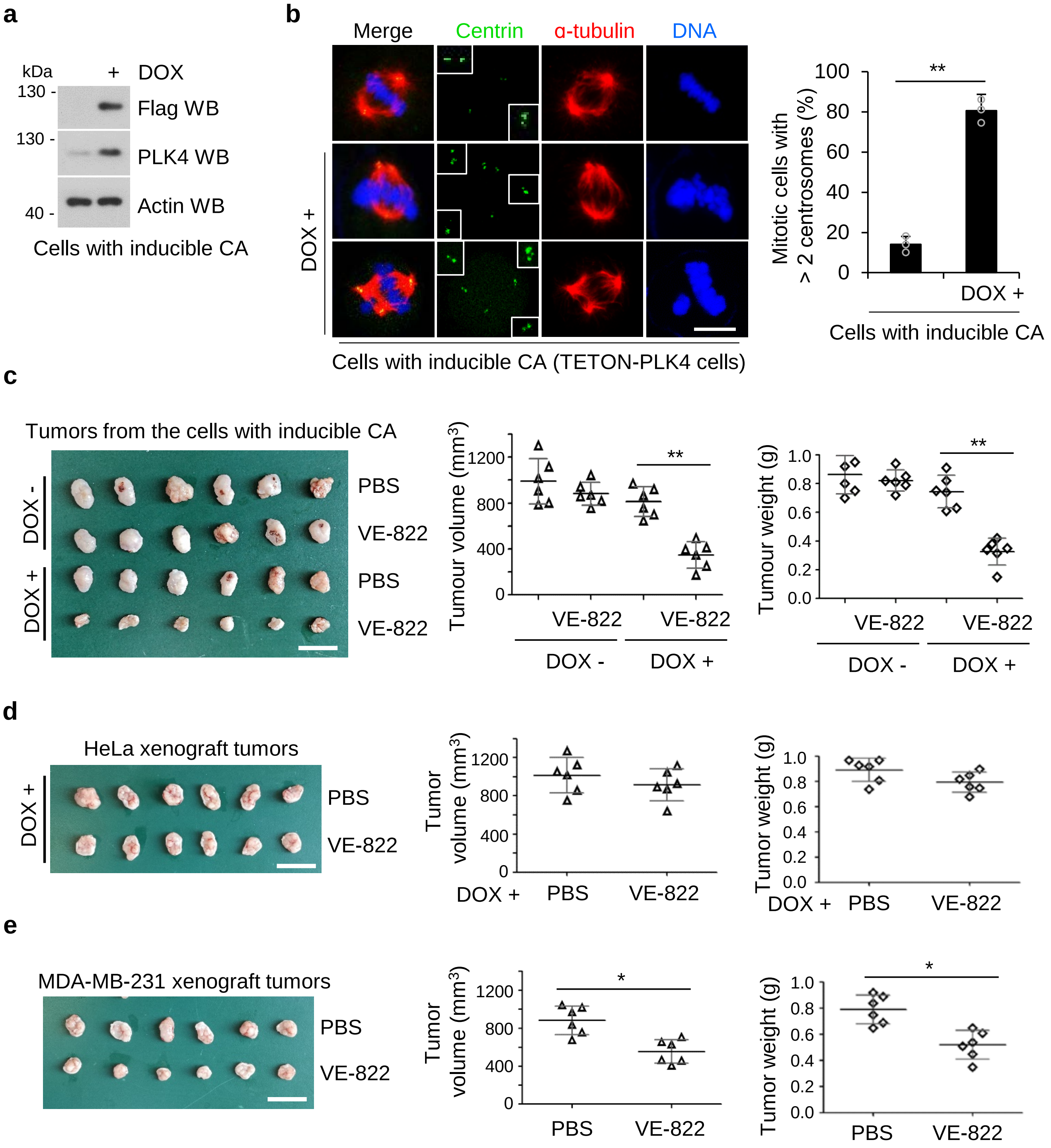

Figure S9, Related to Figure 6. VE-822 inhibits the growth of tumors with high-frequency of centrosome amplification. (a, b) The stable monoclonal cell line was treated with or without 2μg/mL doxycycline (DOX) for 48 h. The lysates were immunoblotted with antibodies against Flag, PLK4, and β-actin. (b) After treatment with DOX, TETON-PLK4 stable cell lines showed centrosome amplification (> 2 centrosomes). Histogram showing the percentage of > 2 centrosomes in mitotic cells. Data represent the mean ± SD of three times of independent experiments. For each experimental condition, 100-138 cells were counted, and three independent experiments were performed. Two-tailed student t test *p* values: $p = 0.0022$. Scale bar, 10 μm. Spindle poles, centrioles, and DNA were co-stained with α-tubulin, centrin-2, and DAPI, respectively. (c, d, and e) Xenograft experiment with TETON-PLK4 cells (c), HeLa cells (d), or MDA-MB-231 cells (e) were described in the Methods section. Tumors were collected and photographed (scale bar, 2 cm). Quantification of average tumor volumes and weights. Data represent the mean ± SD (n =6). (c) Two-tailed student t test *p* values (from left to right): $p = 0.0002$ and < 0.00001. (e) Two-tailed student t test *p* values (from left to right): $p = 0.0144$ and 0.0161. Source data are provided as a Source Data file.

Supplementary data Figure 10

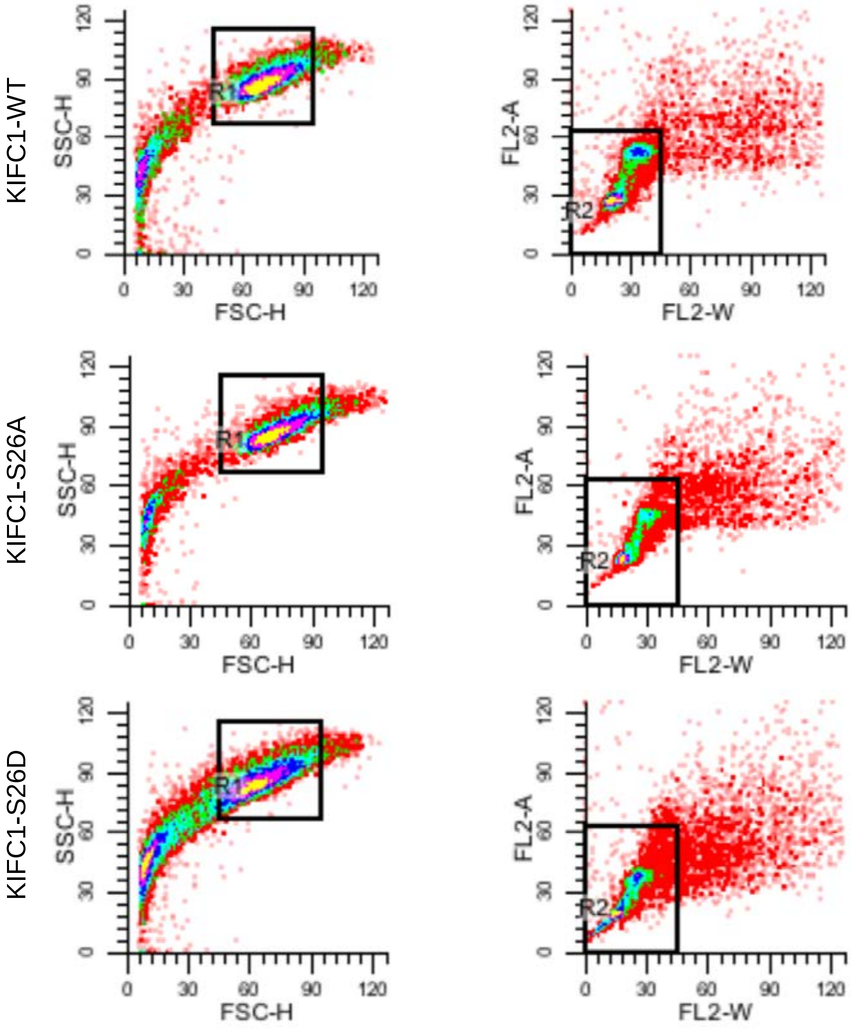

Figure S10. All FACS sequential gating strategies for figures S8a.
