# Supplementary material for: The ATM and ATR kinases regulate centrosome clustering and tumor recurrence by targeting KIFC1 phosphorylation
Source: Nat Commun. 2021 Jan 4;12:20. doi: 10.1038/s41467-020-20208-x (PMC7782532; doi:10.1038/s41467-020-20208-x)
Supplement: Supplementary file 3 — Reporting Summary [file 41467_2020_20208_MOESM3_ESM.pdf]

nature research

Corresponding author(s): Shengping Zhang and Chuangui Wang
Last updated by author(s): Nov 10, 2020

Reporting Summary

Nature Research wishes to improve the reproducibility of the work that we publish. This form provides structure for consistency and transparency in reporting. For further information on Nature Research policies, see our Editorial Policies and the Editorial Policy Checklist.

Statistics

For all statistical analyses, confirm that the following items are present in the figure legend, table legend, main text, or Methods section.

n/a	Confirmed	
☐	☒	The exact sample size (n) for each experimental group/condition, given as a discrete number and unit of measurement
☐	☒	A statement on whether measurements were taken from distinct samples or whether the same sample was measured repeatedly
☐	☒	The statistical test(s) used AND whether they are one- or two-sided<br>*Only common tests should be described solely by name; describe more complex techniques in the Methods section.*
☒	☐	A description of all covariates tested
☒	☐	A description of any assumptions or corrections, such as tests of normality and adjustment for multiple comparisons
☐	☒	A full description of the statistical parameters including central tendency (e.g. means) or other basic estimates (e.g. regression coefficient) AND variation (e.g. standard deviation) or associated estimates of uncertainty (e.g. confidence intervals)
☐	☒	For null hypothesis testing, the test statistic (e.g. F, t, r) with confidence intervals, effect sizes, degrees of freedom and P value noted<br>*Give P values as exact values whenever suitable.*
☒	☐	For Bayesian analysis, information on the choice of priors and Markov chain Monte Carlo settings
☒	☐	For hierarchical and complex designs, identification of the appropriate level for tests and full reporting of outcomes
☒	☐	Estimates of effect sizes (e.g. Cohen's d, Pearson's r), indicating how they were calculated

Our web collection on statistics for biologists contains articles on many of the points above.

Software and code

Policy information about availability of computer code

Data collection	No software was used.
Data analysis	Prism 5 for windows 5.01v, Image J. 1.48v, ModFit LT3.2

For manuscripts utilizing custom algorithms or software that are central to the research but not yet described in published literature, software must be made available to editors and reviewers. We strongly encourage code deposition in a community repository (e.g. GitHub). See the Nature Research guidelines for submitting code & software for further information.

Data

Policy information about availability of data

All manuscripts must include a data availability statement. This statement should provide the following information, where applicable:
- Accession codes, unique identifiers, or web links for publicly available datasets
- A list of figures that have associated raw data
- A description of any restrictions on data availability

No datasets were generated or analyzed during the current study. Other raw data are available on request.

Field-specific reporting

Please select the one below that is the best fit for your research. If you are not sure, read the appropriate sections before making your selection.

☒ Life sciences ☐ Behavioural & social sciences ☐ Ecological, evolutionary & environmental sciences

For a reference copy of the document with all sections, see nature.com/documents/nr-reporting-summary-flat.pdf

Life sciences study design

All studies must disclose on these points even when the disclosure is negative.

Sample size	Group size was determined based on the results of preliminary experiments and referenced "Loss of KLF14 triggers centrosome amplification and tumorigenesis. Guangjian Fan et at. Nature Communications. 2015." No statistical method was used to predetermine sample size in animal studies.
Data exclusions	No data was excluded.
Replication	All the results haven been biologically repeated three times or more. All attempts at replication were successful.
Randomization	Yes, all the animal were randomly to be grouped in all the experiments.
Blinding	The group design and outcome analysis were not performed in a blinded manner, because this is a routine chemistry experiment which is hard to expected. All these experiments have been recorded daily in the lab notebook and all the raw data have been securely stored in the lab.

Reporting for specific materials, systems and methods

We require information from authors about some types of materials, experimental systems and methods used in many studies. Here, indicate whether each material, system or method listed is relevant to your study. If you are not sure if a list item applies to your research, read the appropriate section before selecting a response.

Materials & experimental systems

n/a	Involved in the study	
☐	☒	Antibodies
☐	☒	Eukaryotic cell lines
☒	☐	Palaeontology and archaeology
☐	☒	Animals and other organisms
☐	☒	Human research participants
☒	☐	Clinical data
☒	☐	Dual use research of concern

Methods

n/a	Involved in the study	
☒	☐	ChIP-seq
☐	☒	Flow cytometry
☒	☐	MRI-based neuroimaging

Antibodies

Antibodies used

Anti-KIFC1-Rabbit, HPA055997, Lot: R74598, Sigma-Aldrich;
Anti-KIFC1-Rabbit, ab172620, Lot: GR3259790-1, Abcam;
Anti-ATM-Rabbit, ab32420, Lot: GR198716-38, Abcam;
Anti-ATR-Rabbit, YT0416, Lot: B1601, Immunoway;
Anti- p-ATM-Rabbit, #5883, D6H9, CST;
Anti-Lamin-A/C-Rabbit, ab108922, Lot: GR259331-18, Abcam;
Anti-Flag-Mouse, F1804, Lot: SLBT7654, Sigma-Aldrich;
Anti-phospho-ATM/ATR substrate (S/TQ)-Rabbit, #9607, CST;
Anti-γH2AX-Mouse, ab26350, Abcam;
Anti-γ-tubulin-Mouse, T5326, Sigma-Aldrich;
Anti-Centrin-Rabbit, C7736, Sigma-Aldrich;
Anti-α-tubulin-Mouse, T5199, Sigma-Aldrich;
Anti-PARP-Rabbit, 13371-1-AP, Proteintech;
Anti- activated-caspase-3-Rabbit, BS7004, Bioworld Technology;
Anti-β-actin-Mouse, A5316, Sigma-Aldrich;
Anti-KIFC1 S26p, Bingtai Bio, Custom antibody
Alexa Fluor 568 Goat anti-Mouse IgG (H+L) Cross-Adsorbed Secondary Antibody, A11004, Lot: 1862187, Invitrogen;
Alexa FluorTM 488 AffiniPure Goat Anti-Mouse IgG (H+L), 115-545-003, Lot: 146108, Jackson ImmunoResearch;
Rhodamine RedTM-X (RRX) AffiniPure Goat Anti-Rabbit IgG (H+L), 111-295-003, Lot: 129297, Jackson ImmunoResearch;
Alexa FluorTM 488 AffiniPure Goat Anti-Rabbit IgG (H+L), 111-545-003, Lot:146644, Jackson ImmunoResearch;

Validation	All the antibodies can be validated in the published study and in our Western blot assays.

Eukaryotic cell lines

Policy information about cell lines

Cell line source(s)	293T, HeLa, MDA-MB-231, HCT 116, MCF-10A, BT549, H1299, and OPM-2 cells
Authentication	293T, HeLa, MDA-MB-231, HCT 116, MCF-10A, BT549, and H1299 were purchased from ATCC. OPM-2 was purchased from Biovector Science Lab, Inc, and was not authenticated. Currently, It is under STR examination by Sangon Biotech. (Shanghai, China).
Mycoplasma contamination	It is negative for mycoplasma.
Commonly misidentified lines (See ICLAC register)	No commonly misidentified cell lines were used in the study.

Animals and other organisms

Policy information about studies involving animals; ARRIVE guidelines recommended for reporting animal research

Laboratory animals	BALB/c nude mice, Female, 6-8 weeks old
Wild animals	The study did not involve wild animals.
Field-collected samples	The study did not involve samples collected from the field.
Ethics oversight	Animals were maintained according to the ethical and scientific standards of Animal Center at East China Normal University.

Note that full information on the approval of the study protocol must also be provided in the manuscript.

Human research participants

Policy information about studies involving human research participants

Population characteristics	Study participants are aged 18-75 at recruitment between 2019 and 2024. The patients were diagnosed of cancer with histopathology.
Recruitment	Study participants were recruited through Shanghai LIWEN Biotech Co., Ltd<br>The inclusion criteria are:<br>1. cancer patients diagosed with histopathology, aged 18-75 years old;<br>2. Fresh tumor samples can be obtained through surgury, centesis or pleural effusion and ascites;<br>3. Have tumors meeting RECIST 1.1 standard and the clinical efficacy can be evaluated using CT/MRI;<br>4. ECOG score <=2, expected survival >=3 months;<br>5. Subjects are voluntary to be involved in this trial and sign informed consent form; have good compliance.<br>The following are criteria for exclusion of patients:<br>1. Patients who have incomplete clinical information;<br>2. Patients who undergo unplanned second surgery after first surgery;<br>3. Patients who are discovered to have second cancer during treatment process, which may interfere this trial.<br>The collected tumor samples of enrolled patients were used to build biobank and tumor animal models with consent from patients.
Ethics oversight	The protocol was approved by the China Ethics Committee of Registering Clinical Trials.

Note that full information on the approval of the study protocol must also be provided in the manuscript.

Flow Cytometry

Plots

Confirm that:

☒ The axis labels state the marker and fluorochrome used (e.g. CD4-FITC).

☒ The axis scales are clearly visible. Include numbers along axes only for bottom left plot of group (a 'group' is an analysis of identical markers).

☒ All plots are contour plots with outliers or pseudocolor plots.

☒ A numerical value for number of cells or percentage (with statistics) is provided.

Methodology

Sample preparation	Cells were trysinized, harvested, and fixed with 0.5ml 70% EtOH (pre-cooled to -20°C overnight) for 24 h. After fixation, Cells

Sample preparation	were spinned down for 2min at 2,000 rpm, resuspended in 0.5ml PBS containing 10µg/ml RNase A and 20µg/ml PI (Propidium iodide), transfered to FACS tubes, and incubated at room temperature in the dark for 30 min. Ready for FACS.
Instrument	BD LSRFortessa
Software	ModFit LT3.2
Cell population abundance	The instrument counts 10,000 cells autonomously.
Gating strategy	First plot gating (FSC-A/SS-H) for live cells, then second plot (FSC-A/FSC-H) for single and then only for PI positive cells.

☒ Tick this box to confirm that a figure exemplifying the gating strategy is provided in the Supplementary Information.
